# Cerebral Microdialysis for Protein Biomarker Monitoring in the Neurointensive Care Setting – A Technical Approach

**DOI:** 10.3389/fneur.2014.00245

**Published:** 2014-12-03

**Authors:** Lars Hillered, Andreas P. Dahlin, Fredrik Clausen, Jiangtao Chu, Jonas Bergquist, Klas Hjort, Per Enblad, Anders Lewén

**Affiliations:** ^1^Division of Neurosurgery, Department of Neuroscience, Uppsala University, Uppsala, Sweden; ^2^Division of Microsystems Technology, Department of Engineering Sciences, Uppsala University, Uppsala, Sweden; ^3^Analytical Chemistry, Department of Chemistry-BMC and SciLifeLab, Uppsala University, Uppsala, Sweden

**Keywords:** microdialysis, catheter performance, acute brain injury, neurointensive care, protein biomarkers, intracranial pressure, biofouling, inflammation

## Abstract

Cerebral microdialysis (MD) was introduced as a neurochemical monitoring method in the early 1990s and is currently widely used for the sampling of low molecular weight molecules, signaling energy crisis, and cellular distress in the neurointensive care (NIC) setting. There is a growing interest in MD for harvesting of intracerebral protein biomarkers of secondary injury mechanisms in acute traumatic and neurovascular brain injury in the NIC community. The initial enthusiasm over the opportunity to sample protein biomarkers with high molecular weight cut-off MD catheters has dampened somewhat with the emerging realization of inherent methodological problems including protein–protein interaction, protein adhesion, and biofouling, causing an unstable *in vivo* performance (i.e., fluid recovery and extraction efficiency) of the MD catheter. This review will focus on the results of a multidisciplinary collaborative effort, within the Uppsala Berzelii Centre for Neurodiagnostics during the past several years, to study the features of the complex process of high molecular weight cut-off MD for protein biomarkers. This research has led to new methodology showing robust *in vivo* performance with optimized fluid recovery and improved extraction efficiency, allowing for more accurate biomarker monitoring. In combination with evolving analytical methodology allowing for multiplex biomarker analysis in ultra-small MD samples, a new opportunity opens up for high-resolution temporal mapping of secondary injury cascades, such as neuroinflammation and other cell injury reactions directly in the injured human brain. Such data may provide an important basis for improved characterization of complex injuries, e.g., traumatic and neurovascular brain injury, and help in defining targets and treatment windows for neuroprotective drug development.

## Introduction

Cerebral microdialysis (MD) is currently widely used for the sampling of low molecular weight (<200 Da) biomarkers of energy crisis and cellular distress in the neurointensive care (NIC) setting (1). There is an emerging interest in MD for the sampling of protein-based biomarkers of secondary injury mechanisms in NIC patients with acute traumatic and neurovascular brain injury (2–4). Evolving analytical methodology allowing for multiplex biomarker analysis in 1–25 μL individual samples opens a new possibility for temporal mapping of complex secondary injury cascades, such as inflammation and cell-specific injury components. In this context, recent MD studies in NIC patients have presented temporal patterns of inflammatory biomarkers (5–9). The study by Helmy et al. on multiple (*n* = 42) inflammatory biomarkers also supports the notion that the innate immune system of the brain is activated early after traumatic brain injury (TBI), making MD an attractive focal sampling method for e.g., cytokines, chemokines, and neurotrophic factors (5, 10), as a complement to global biomarker analysis in ventricular cerebrospinal fluid (CSF) (*vide infra*).

Numerous *in vitro* studies have revealed that MD protein biomarker sampling is more complex than traditional low molecular weight biomarker sampling, involving protein–protein interaction, protein–surface interaction, and biofouling [for references, see Ref. (11, 12)]. By using nano liquid chromatography (nanoLC) in combination with tandem mass spectrometry (MS/MS), we showed that the proteins adsorbed onto the MD membrane may be lost to biomarker analysis in the dialysate since they are prevented from crossing the MD membrane (13). In addition, there is concern that changes in intracranial pressure (ICP), a common phenomenon in acute brain injury patients, may influence MD catheter performance *in vivo*. Thus, Helmy et al. [(5), Figure 2 in Supplementary Material] found a significant correlation between ICP and fluid recovery (FR; the percentage of perfusate collected after passage through the catheter) with crystalloid perfusion medium in TBI patients that was abolished by the addition of 3% human albumin, suggesting that the colloid osmotic pressure of the perfusate is important for optimal MD catheter performance. These results have inspired research of the mechanisms and challenges involved with MD protein biomarker sampling.

As our published *in vitro* studies in this area show, using large dextran colloids in the MD perfusate stabilizes the pressures within the MD system, leading to FR values close to 100%, which is the target for comparative studies. Also, by dynamically modifying the surfaces of the membrane and the inner tubing of the MD catheter by self-assembly of amphiphilic tri-block polymer coating (Pluronic^®^ F-127), we were able to decrease the protein adsorption and increase precision in FR, improving extraction efficiency (EE, a.k.a. relative recovery; i.e., the concentration of an analyte in the dialyzate divided by the concentration of the same analyte in the bulk sample) for some proteins in human ventricular CSF (11). By using nanoLC MS/MS analysis, we showed that protein adsorption to the MD membrane was reduced by 33% in surface-modified compared to control catheters (14). Our hypothesis is that the combination of large dextran colloids in the MD perfusate and the lowering of protein adsorption to the MD membrane and tubing will reduce biofouling and improve FR and protein biomarker EE, thereby increasing the overall robustness of MD catheter performance.

This hypothesis was recently tested when our refined MD methodology was validated in a clinically relevant model of acute brain injury (15). The results supported our hypothesis by showing that MD catheters with surface modification and Dextran 500 (kDa) in the perfusate had a more stable FR close to 100% that was insensitive to changes in ICP, no significant difference in the EE of low molecular weight biomarkers, and an improved and more homogenous EE for protein biomarkers in response to the intervention compared to naïve catheters (16).

The purpose of this short review is to describe in more detail the background *in vitro* work leading up to this refined methodology. In addition, we discuss the potential of MD for pattern mapping and monitoring of a large number of protein biomarkers, using novel multiplex analytical methodology in ultra-small (1–5 μL) MD samples in the NIC setting.

## Protein Biomarker Sampling with Microdialysis – A Complex Task

During the last several years, the search for novel protein biomarkers of neurodegenerative diseases has escalated. The priority has been to examine the proteomics of CSF, due to its direct connection and neurochemical exchange with the brain. CSF is a colorless body fluid that contains very few cells (0–4 cells/μL), low protein concentration (0.05–0.8 mg/mL), and a salt concentration similar to blood (17). However, the analytical challenge lies in the fact that the CSF contains a large number of proteins spanning over a concentration range of at least 10 orders of magnitude (18), requiring appropriate and reliable sampling, sample treatment, and detection techniques.

Since its introduction in the 1970s (19), MD has become an established sampling technique routinely used for several decades (20, 21). MD was introduced as a clinical sampling method for neurochemical brain monitoring in NIC patients in the 1990s (22). Being a unique intracerebral sampling tool, MD is currently used in NIC worldwide, mainly as a clinical research tool but also for routine chemical brain monitoring in some neurosurgical centers (1, 23–27). Inherent limitations, including invasiveness, focal sampling (i.e., representativity of MD data), labor intensiveness, and cost have thus far prevented a general breakthrough for routine use of MD in NIC.

Microdialysis is a membrane based *in vivo* sampling technique where a perfusion fluid is continuously flowing inside a hollow fiber membrane and thereby extracting the molecules at the outside environment. The most important parameter in conventional MD is the EE, which describes the overall efficiency of the sampling (20). However, when using large pore membranes (MWCO ≥100 kDa), it is equally important to also control the FR to enable stable, diffusion-driven sampling during the entire experiment/monitoring period, and thereby facilitate correct biological interpretation of the MD data.

The traditional main use of MD has been to collect small hydrophilic molecules, e.g., glucose, lactate, pyruvate, glutamate, glycerol, and urea, from different biological matrices (20). With the introduction of MD membranes with a MWCO of 100 kDa or more, the focus has shifted toward developing MD methods for sampling of larger biomolecules, such as peptides (28, 29), cytokines (2, 12, 30–33), and other proteins (34–38). The studies together strongly suggest that MD is a promising tool for targeted analysis, validation, and monitoring of well described biomarkers in the NIC setting, as a complement to traditional CSF analysis.

When 100 kDa membranes are used for sampling proteins, the reported EEs range between 1 and 5% at perfusion flow rates of 0.5–1.0 μL/min (36). However, more alarmingly, the EE for individual biomarkers has shown to vary extensively despite their similarities in molecular mass (12). The reproducibility and the repeatability have been reported to be poor (11, 38, 39), indicating unreliable and non-robust methodology. Li et al. (40) listed five challenges that must be considered when sampling proteins with MD. Firstly, proteins are often present in low concentrations and a highly dynamic concentration range in CSF. Secondly, the size of the proteins decreases the diffusion velocity. Thirdly, membranes with large pores are more sensitive to different pressures and perfusion fluid may be lost or gained in an uncontrolled manner. Fourthly, proteins instantly and dynamically adsorb onto the membrane and tubing of the MD catheter, leading to catheter fouling. Finally, MD generates small sample volumes containing low concentration of proteins, which could be problematic to analyze properly. It is important to have maximum control over all five challenges to facilitate the transfer of a new MD method from *in vitro* to *in vivo* application. By using a representative CSF sample material, sampling at 37°C, at a controlled ambient pressure, etc., the goal of mimicking the CSF conditions *in vivo* in terms of sample properties, the concentration, and diffusion properties of the proteins may be achieved.

## Description of *In vitro* Model

The aim of an *in vitro* system is to test a hypothesis in a controlled environment and then to use that knowledge and apply it on the *in vivo* model. The more similar one can design the *in vitro* system the more accurate an extrapolation will be to the *in vivo* system. The least complex *in vitro* system would be one catheter in a sample containing one protein dissolved in water, sampled in room temperature, atmospheric pressure, and with a perfusate consisting of water. This is a hydrodynamically unstable system that will give very varying results with respect to FR and EE. The most complex *in vitro* set up would be a multi catheter system placed in a microchamber containing a biological sample. The sample should be pressurized to 5–50 mmHg and with high level of accuracy. The MD sampling should be performed in 37°C with controlled pH.

**Figure 1:**
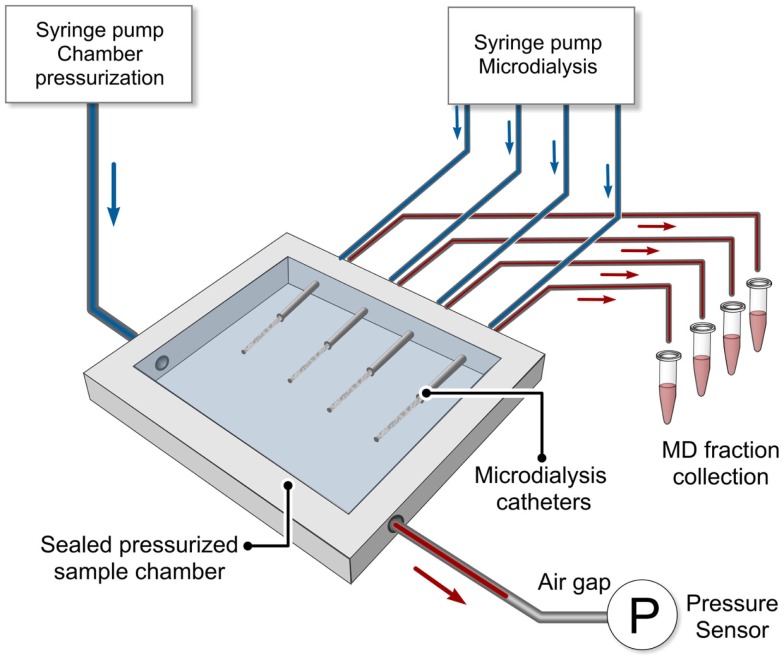
**Schematic drawing of a pressurized *in vitro* MD sampling system is shown**. The chamber held four MD catheters and was used to study the impact of different pressures present in a MD sampling system on fluid recovery (42). Pressure parameters tested included perfusion flow rate, colloid osmotic additives in both perfusate and sample, and static outside pressure. The precision of the system was <0.5 mmHg and pressures could be set accurately in order to simulate different NIC conditions [adopted from Ref. (42)].

Parameters such as temperature, ion strength, and pH can easily be set in *in vitro* systems. However, when using membranes with MWCO ≥100 kDa, it is also important to be able to control the static outside pressure exerted by the sample. This parameter is most often overlooked since the outside static pressure often remains on a constant level throughout the experiment. A pressurized *in vitro* experiment is also more complex to carry out since it requires additional instrumentation and equipment. However, since human ICP may differ between 2 and 15 mmHg in healthy humans (41) to more than 50 mmHg in NIC patients; such changes may have a significant impact on the FR and hence the EE. Therefore, we decided to develop a more complex *in vitro* model. Figure 1 shows a schematic drawing of a pressurized *in vitro* sampling system. The chamber holds four MD catheters and was used to study the impact of different pressures present in a MD sampling system on FR (42). The pressure parameters that were tested included perfusion flow rate, colloid osmotic additives in perfusate, and sample, as well as static outside pressure. The precision of the system was <0.5 mmHg, and pressures could be set accurately in order to simulate different NIC conditions.

## Fluid Balance and Sensitivity to Changes in External Pressure

**Figure 2:**
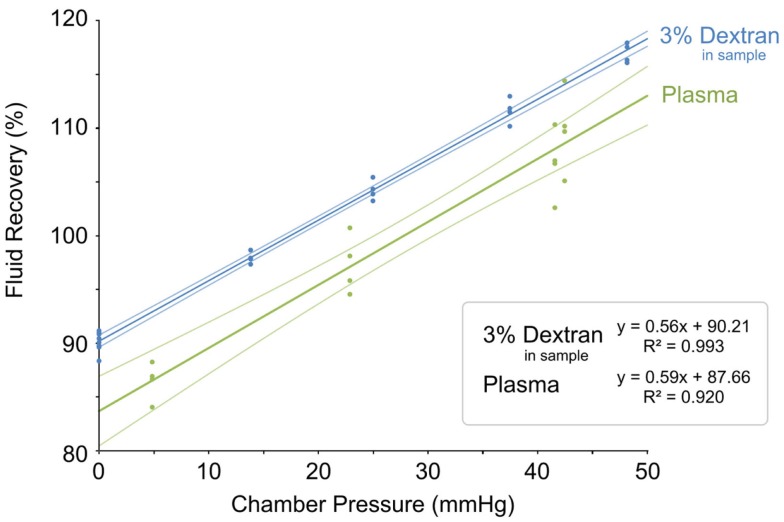
**Fluid recovery plotted as a function of external sample pressure**. *In vitro* MD test fluid recovery plotted as a function of external sample pressure in dextran sample (blue) and plasma sample (green) (42). Reprinted with permission from Chu (42). Copyright 2014 Springer Science Media.

It was found that increased external pressure also increased the FR (42), which was in agreement with a previous study (43). However, the increase in FR was also highly dependent on the perfusion fluid flow rate and the concentration of colloid osmotic agent in both the perfusate and the sample. The FR increased faster with decreased perfusion flow rate. For example, FR increased from 108% (0 mmHg) to approximately 800% (50 mmHg) when a perfusion flow rate of 0.5 μL/min was used in a system without colloid osmotic agents. When the flow rate was increased to 1 μL/min, the FR increase was less dramatic, from 72% (0 mmHg) to approximately 500% (50 mmHg). The *in vitro* MD system was very sensitive to pressure changes, but the addition of colloids in the perfusion fluid resulted in a much more stable *in vitro* system where FR increased from around 90% (0 mmHg) up to 200% (50 mmHg) when 3% w/v Dextran 500 (kDa) was used in the perfusate. Furthermore, the *in vitro* system was more stable and less sensitive to external pressure changes when colloids were added in both the perfusate and in the sample chamber. FR increased from 90% (0 mmHg) to 117% (50 mmHg) when 3% w/v Dextran 500 (kDa) was added to the sample (Figure 2). The findings underline the importance of controlling the external, hydraulic, and osmotic pressures during *in vitro* MD, especially when mimicking MD sampling applications with elevated pressures, e.g., intracranial hypertension.

In addition, it is pertinent to control the fluid balance. High MWCO MD membranes (≥100 kDa) are ultra-filtration membranes and more sensitive to different pressures, meaning that their fluid characteristics may move from the traditional dialysis area into ultra-filtration, where fluid flow has to be considered, and diffusive transport may not always dominate the molecular transport. It is therefore advantageous to strive for a FR of 100% (i.e., the volume pumped into the MD catheter should also exit) to secure a diffusion-driven dialysis sampling to provide time resolved samples that are collected with minimal effect on the microenvironment. If the FR is smaller than 100%, perfusion fluid will leak out from the membrane and dilute the microenvironment. If the FR is larger than 100%, the opposite effect will take place, i.e., the flux of water from the sample to the dialyzate through the MD membrane, may result in a higher concentration of larger molecules at the vicinity of the MD membrane. In the course of a disease process with dynamic ICP, FR may fluctuate causing unstable MD catheter performance. One way to stabilize fluidic flow is to increase the osmotic pressure of the perfusate. This may be done by adding a colloid such as albumin to the perfusate (12, 33–35). However, albumin is not compatible with proteomic applications, which use LC in combination with MS-based detection since any added albumin would completely dominate the sample loading in both the LC and the MS signal. As an alternative to albumin, dextrans with a molecular mass of 60–70 kDa, have been used to increase the osmotic pressure. (30, 37, 38, 40, 44, 45). Dextrans, which is the collective name for large (1 kDa – 2 MDa) and neutral polysaccharides, can easily be removed prior to separation and detection and do thereby not affect the MS signal.

**Figure 3:**
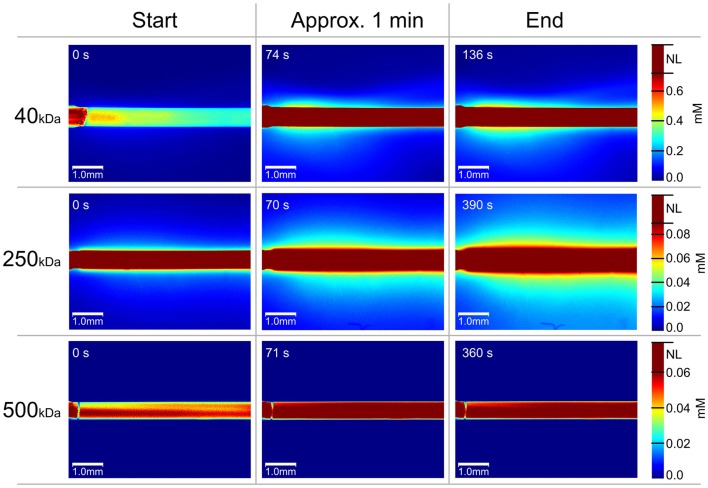
**Temporal evolution of MD catheter dextran leakage pattern is shown**. All images are color coded according to the pixel color-dextran molecular concentration linearity, to reveal quantitative information on the dextran leakage. The scale bars on the right show the dextran molar concentration (millimolar) depending on the pixel color. The pixels, which have color over the linear zone are all marked as non-linear (“NL”), which is displayed in dark red. Hundred kilodaltons of MWCO membranes are used (100 kDa Brain Microdialysis Catheter, M Dialysis AB). For Dextran 40 (kDa), three image timings are: 0 s, 1 min 14 s, 2 min 18 s; for Dextran 250 (kDa), the image timings are: 0 s, 1 min 10 s, 6 min 30 s; and for Dextran 500 (kDa), the image timings are: 0 s, 1 min 11 s, 6 min. Reprinted with permission from Chu (46). Copyright 2014 Springer Science Media.

When using membranes with MWCO ≥100 kDa, dextrans with average size of 500 kDa should be used in order to avoid leakage to the sample (46). In a recent study, an *in vitro* MD microchamber was manufactured with dimensions of a standard microscope slide (27 mm × 79 mm) and ability to hold one MD catheter. The high MWCO catheter (100 kDa Brain Microdialysis Catheter, M Dialysis AB) was perfused with differently sized fluorescent dextrans with the aim to investigate leakage patterns. Leakage patterns, due to MD phenomena such as bubble formation, cracked membranes, inward ultra-filtration flux, and diffusion, were analyzed in real time using a fluorescence microscope. A semi-quantitative analysis was performed on perfusates containing dextrans with average sizes of 40, 250, and 500 kDa. It was found that both Dextran 40 (kDa) and Dextran 250 (kDa) leaked extensively despite the 100 kDa MWCO membrane (Figure 3). The explanation may be that the MWCO of a membrane is equal to the MW at which 80% of the molecules are prevented from flux through the dialysis membrane (47), meaning that the MWCO is not an absolute measure of the pore size of the membranes. Another reason may be that the declared MW of dextran is based on an approximation of a distribution of MWs in the solution, meaning that dextran molecules with both considerably larger and lower MWs are present. For example, a certified, 270 kDa dextran standard has a MW-dispersion ranging from 13 kDa to 6.4 MDa and around 14% of the dextrans in a 270 kDa dextran standard are smaller than 100 kDa. As can be seen in Figure 3, no leakage was observed when Dextran 500 (kDa) was used as perfusate. We therefore recommend that 100 kDa membrane MD catheter users switch to Dextran 500 (kDa) rather than using Dextran 60 (kDa) in the perfusate.

## Intracranial Pressure Sensitivity *In vivo*

There is concern that changes in ICP, a common phenomenon in acute brain injury patients, may influence the *in vivo* performance of 100 kDa MWCO catheters widely used in the NIC setting. This was based on a study by Helmy et al. [(5), Figure 2 in Supplementary Material] who found a significant correlation between ICP and FR with crystalloid perfusion medium in TBI patients that was alleviated by the addition of 3% human albumin, suggesting that the colloid osmotic pressure of the perfusate is important for optimal MD catheter performance.

**Figure 4:**
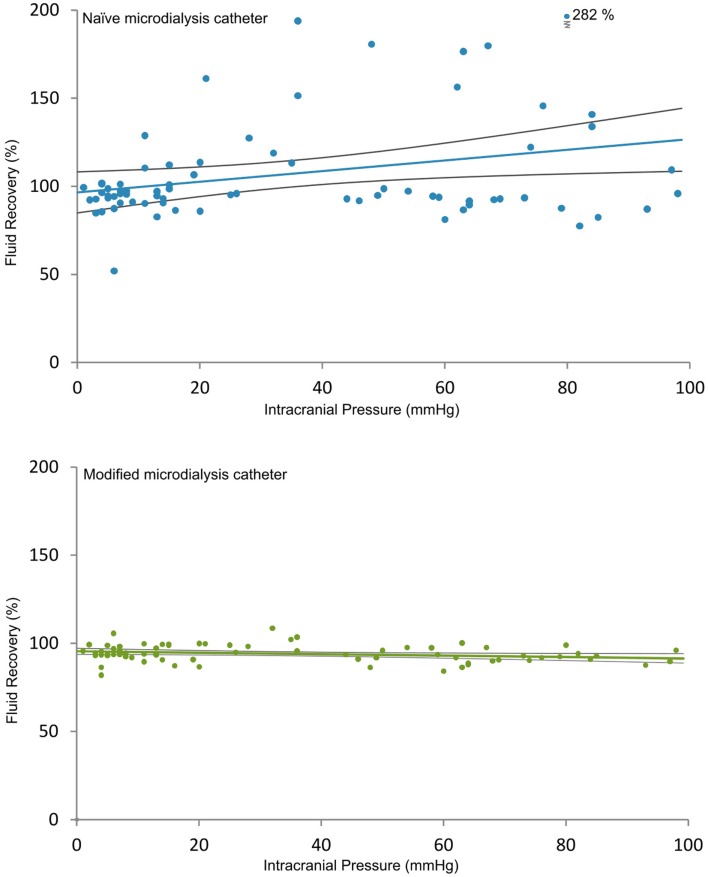
**Relation between fluid recovery and ICP for naïve and modified MD catheters is shown**. The two diagrams show the relation between FR and ICP for the naïve (upper) and the modified (lower) MD catheters (100 kDa Brain Microdialysis Catheter, M Dialysis AB), both perfused (1 μL/min) with Perfusion Fluid CNS with in-house addition of 3% Dextran 500 (kDa). Each circle is the FR of an individual MD vial plotted against the mean ICP at the end of each corresponding 20-min period. A linear regression line is plotted with 95% confidence interval. Upper panel shows a significant positive correlation between FR and ICP in naïve MD catheters with a high degree of variability (*r* = 0.30, *p* = 0.02, Standard Error of Estimate = 32.3). Lower panel shows that this correlation switched to a weak negative relation in modified catheters with markedly reduced scatter (*r* = −0.04, *p* = 0.04, Standard Error of Estimate = 4.8), suggesting a more stable MD performance as a result of surface modification (16). Reprinted with permission from Dahlin et al. (16). Copyright American Chemical Society.

In a recent study in a porcine model of acute brain injury caused by a gradual elevation of ICP leading to brain death (15), we tested the FR of naïve and adjacent MD catheters (100 kDa Brain Microdialysis Catheter, M Dialysis AB, Stockholm, Sweden) surface coated with Pluronic F-127. Both catheters were perfused (1 μL/min) with Perfusion Fluid CNS (M Dialysis) with a 3% in-house addition of Dextran 500 (kDa) (16). We found a significant positive correlation between FR and ICP in naïve MD catheters (Figure 4, upper panel) with a high degree of variability (*r* = 0.30, *p* = 0.02, Standard Error of Estimate = 32.3). However, in surface-modified catheters (Figure 4, lower panel), the FR dependency of ICP was virtually abolished with markedly reduced scatter among data points (*r* = −0.04, *p* = 0.04, Standard Error of Estimate = 4.8), suggesting a more stable MD performance as a result of surface modification in combination with Dextran 500 (kDa). We believe that this improvement in the FR performance of the MD catheter in a clinically relevant range of ICP and CPP levels is instrumental for a stable and diffusion-driven biomarker sampling in the NIC setting. Furthermore, the use of Dextran 500 (kDa) in the perfusate may improve the safety of clinical MD based on the concern that leakage of Dextran 70 (kDa) from the MD catheter may cause an inflammatory reaction in the tissue, which may be prevented by the use of Dextran 500 (kDa) as colloid (8, 48).

It is our working hypothesis that a stable FR close to 100% is instrumental for a purely diffusion-driven and stable EE as supported by our *in vivo* data showing a more homogenous biomarker response to intracranial hypertension in modified compared to naïve MD catheters (16). However, EE in modified catheters improved for some but not for all proteins, suggesting that the effect of ICP on EE is a complex issue. For example, elevated ICP may lead to increased protein levels in the interstitial space related to, e.g., cell damage and BBB leakage, leading to changes in protein interactions that may influence EE for individual proteins in a complex fashion. To better understand the precise mechanisms involved, we are in the process of studying the effect of ICP on EE in our pressurized *in vitro* sampling model described above (42). Such knowledge may provide a basis for further refinement and optimization of the MD method.

## Biofouling

**Figure 5:**
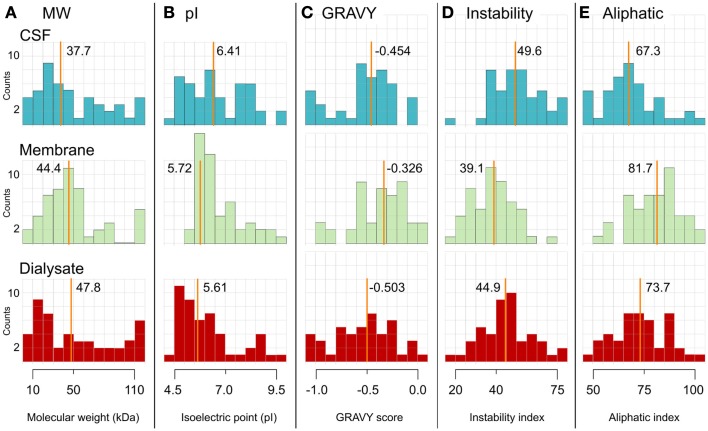
**Distribution histograms for proteins in CSF, on the MD membrane, and in the dialyzate are shown**. Protein distribution histograms for protein properties of the proteins identified in CSF (top, blue), adsorbed to the MD membrane (middle, green) and in the dialyzate (bottom, red). The 11 proteins found in all samples are excluded. The proteins are divided in fractions depending on their calculated number of each property. The investigated properties are **(A)** molecular weight, **(B)** isoelectric point, **(C)** GRAVY score, **(D)** instability index, and **(E)** aliphatic index. The *y*-axis is the number of proteins found within the range (*x*-axis). The line in each histogram represents the median value, which is also written next to the line, *n* = 59 for CSF, *n* = 63 for membrane, and *n* = 59 for dialyzate. The proteins adsorbed onto the membrane had a significantly lower instability index (i.e., more stable), were more hydrophobic (i.e., higher GRAVY score), and were more thermostable (i.e., higher aliphatic index), compared to the proteins in the CSF and the dialyzate. The results suggest that proteins may escape detection because they are adsorbed onto the MD membranes and thereby prevented from passing the membrane into the dialyzate [(13), Figure S1 in Supplementary Material]. Reprinted with permission from Wetterhall et al. (13). Copyright 2014 Elsevier.

As always when a foreign material is inserted into a living organism or biologically active sample, a response will occur. The formation of a protein layer due to adsorption is considered to be the first step in the acute biological response to foreign material (49). This layer is the foundation for further processes, which eventually lead to protein fouling, inflammatory reactions, and encapsulation of the membrane (50, 51). The protein adsorption process is very complex and highly dynamic (52), implying that the protein composition of the surface layer is constantly changing. This affects the properties of the MD catheter membrane by changing the surface chemistry and the membrane pore size (37, 38, 53). Proteins may also be adsorbed to the membrane whereby they escape detection, a discovery recently presented in a qualitative analysis of the protein distribution from a human CSF sample in an *in vitro* MD sampling system (13). A total of 134 different proteins were found in the four analyzed sample compartments (CSF start, Membrane, Dialyzate, CSF end). Most of the identified proteins (*n* = 87) were uniquely found in one sample compartment only. Abundant CSF proteins such as albumin, apolipoproteins, and cystatin C together with plasma proteins such as hemoglobin and fibrinogen were among the 11 proteins that were found in all samples. The proteins adsorbed onto the membrane were significantly more hydrophobic, had a lower instability index, and were more thermostable compared to the proteins in the CSF and the dialyzate (Figure 5). The results suggest that proteins adsorbed onto the MD membranes may escape detection because they are prevented from passing through the membrane into the dialysate. It was concluded that the membrane needs to be examined after sample collection in order to better verify the protein content in the original sample. This is particularly important when searching for new protein biomarkers for neurodegenerative diseases.

One way to reduce and delay protein adsorption is to modify the membrane surface (54). Recently, we presented an alternative approach to modify MD catheters by dynamically attach a tri-block copolymer Poloxamer 407, better known as Pluronic F-127^®^ to the surfaces of the membranes and tubing (11, 14, 16, 53). Pluronic F-127^®^ is a tri-block copolymer consisting two hydrophilic polyethyleneoxid (PEO) chains that sandwich a hydrophobic polypropylenoxide (PPO) unit resulting in a molecular structure PEO_98_–PPO_67_–PEO_98_. In aqueous environment, the hydrophobic PPO-chain is adsorbed to the polymeric and hydrophobic catheter material and the hydrophilic PEO-chains self-assemble into a cilia-like surface that protrude from the hydrophobic membrane and tubing and effectively hinders proteins to reach the surface (55). According to the Food and Drug administration (FDA) guide, Pluronic F-127^®^ is regarded to be an inactive ingredient for different types of preparations such as inhalations, oral solutions, suspensions, ophthalmic, and topical formulations (53). In addition, Pluronic F-127^®^can be sterilized by autoclaving (120°C, 15 min, 1 bar), which potentially facilitates unimpeded transition from *in vitro* to *in vivo* application.

**Figure 6:**
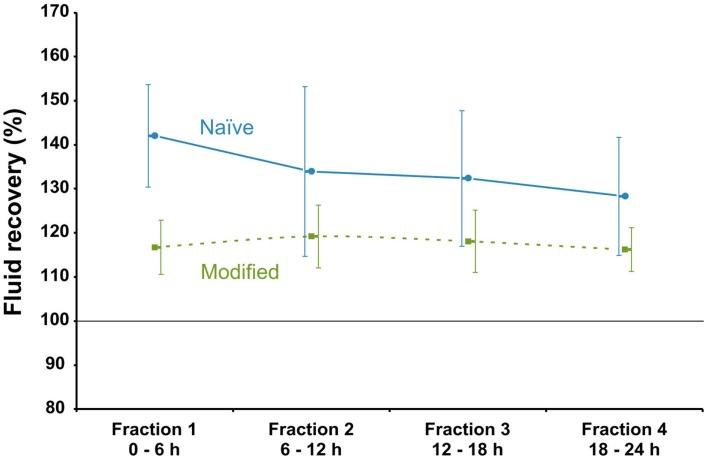
**Fluid recovery as a function of time for naïve and the modified MD catheters *in vitro***. Fluid recovery as a function of fraction order in *in vitro* microdialysis in human ventricular CSF. Perfusion fluid CNS with 3% w/v Dextran 60 was delivered at 0.3 μL/min at 37°C. Blue solid line symbolizes naïve MD catheters and green dotted line surface-modified (Pluronic F-127) MD catheters (100 kDa Brain Microdialysis Catheter, M Dialysis AB). Error bars are based on ±1 SD of four surface-modified and untreated catheters, respectively (11). Reprinted with permission from Dahlin et al. (11). Copyright American Chemical Society.

**Figure 7:**
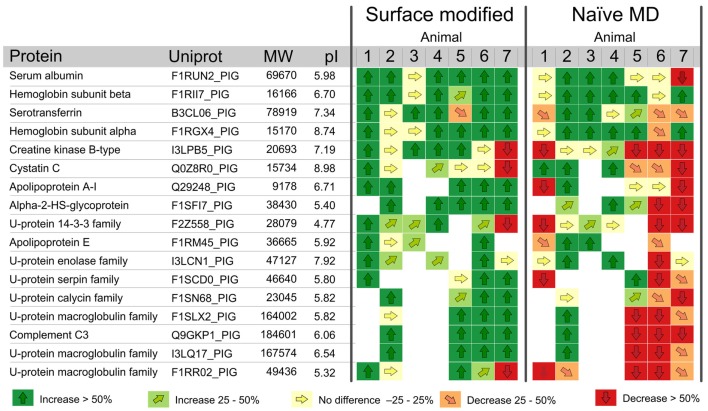
**Protein response to ICP intervention for naïve and modified MD catheters**. Vector diagram showing the response of the 17 most abundant proteins, according to the Mascot score (i.e., the probability that the experimental data set matches the database data) quantified in ≥4 animals, during step-wise intracranial hypertension. Proteins with vectors are relatively quantified. Proteins tagged with “Id” are identified but not quantified, i.e., fulfilling the identification criteria only. Proteins are presented with names, their Uniprot database number (www.uniprot.org), and their response to intervention for surface-modified and naïve catheters. The relative protein amount after intervention (Protein fraction 2, MD fractions 7–12) is compared with the relative protein amount from before intervention (Protein fraction 1, MD fractions 1–6). An arrow pointing straight up (↑) means that the particular protein has increased significantly in concentration by >50% due to the intervention. An arrow pointing straight down (↓) means that the concentration of the protein has decreased significantly by >50% in response to the intervention. Vectors pointing diagonally display either an increase (light green) or a decrease (orange) of 25–50%. These changes are not statistically significant but they show interesting tendencies. Finally, vectors pointing to the right (yellow) range between 0.75 and 1.25 and show no significant difference between the two fractions. *U-protein* means uncharacterized proteins. *White boxes* means that the protein was not identified or quantified in the sample. Thus, a white box is a reflection of the sample composition, not related to MD catheter performance (16). Reprinted with permission from Dahlin et al. (16). Copyright American Chemical Society.

We found that Pluronic F-127^®^ modified MD catheter membranes adsorbed 33% less proteins than untreated MD membranes (14). Surface modification also promoted the EE for some proteins both *in vitro* (11) and *in vivo* (16). Another major advantage with surface-modified MD catheters was a significantly improved FR precision for both *in vivo* (Figure 4) and *in vitro* (Figure 6) MD. The direct result of improved FR precision was more reliable sampling of protein biomarkers (Figure 7), while low molecular weight biomarkers were less affected (16).

## Protein Biomarker Analysis with High Sensitivity and Precision – A Challenge

### Mass spectrometry based analysis of microdialysate protein biomarkers

The combination of low concentrated protein samples and limited sample volumes that MD sampling generates require subsequent separation and the detection method to be both sensitive and selective. Liquid chromatography (LC) in combination with tandem mass spectrometry (MS/MS) is universal, selective, and sensitive. LC-MS is therefore a good choice for the analysis of MD samples. There are several strategies for quantitative MS proteomics (56, 57). One approach for relative quantification is the *isobaric tag for relative and absolute quantification* (iTRAQ^®^), which enables multiplexed quantitative analysis of up to eight samples simultaneously (58). The benefits of using iTRAQ^®^ for MD sample analysis are the possibility to perform differential quantification of complex samples under similar conditions. iTRAQ thereby facilitates direct comparison between the original sample and the dialysate, which is used to determine the EEs for multiple proteins with high sensitivity.

The iTRAQ-MS/MS have been applied to both *in vitro* (11) and *in vivo* (16) MD samples. In the *in vitro* study, 48 proteins were identified and quantified in the dialysate obtained from sampling ventricular CSF during 24 h. Six of the most abundant proteins, with respect to their MASCOT score, were selected for further and more detailed analysis. Albumin, Transferrin, Clusterin, Complement C3, Hemopexin, and Hemoglobin Beta were chosen. The EE for Albumin was on average 17.5 ± 2.4% for the surface treated catheters (*n* = 5) and 17.3 ± 2.4% for the naïve catheters (*n* = 5). Albumin showed no significant difference in EE between surface-modified catheter and naïve catheter. Clusterin and Complement C3 showed no statistically significant difference in EE between Pluronic F-127 coated and naïve catheters. Hemopexin, Transferrin, and Hemoglobin Beta increased significantly in EE when comparing surface coated to naïve catheters (11).

In the *in vivo* study (16), 66 proteins were relatively quantified in one or more animals. However, only proteins that were quantified in ≥4 animals were considered for further evaluation (*n* = 17). These 17 proteins, presented in Figure 7, were used to study the EE performance of the MD catheters. Data were normalized, and thereafter, proteins in MD samples from surface-modified versus naïve MD catheters were analyzed by vector diagrams to illustrate the protein pattern changes in response to the intervention. The vector diagram analysis showed a significantly more homogenous protein pattern in response to the intervention in modified compared to naïve MD catheters. Three naïve catheters (animals 1, 5, and 6) failed to deliver stable FR and also showed large variation in protein response. However, failing catheters was not the only reason for high variability in protein response, which is apparent in animal 7 (Figure 7). The distinctive behaviors of the modified catheters were dual: firstly, they promoted the sampling recovery for most of the proteins found in the dialyzate. Secondly, they presented a more consistent performance. Both features are advantageous, if more consistent sampling performance and higher protein recovery are considered as goals for improving MD *in vivo* performance.

The proteins detected in the proteomic analysis of the MD samples (Figure 7) were used as biomarker models to study the *in vivo* performance of naïve compared to membrane modified MD catheters. However, some of the proteins have functional properties of relevance to brain injury and might even have some potential as future biomarkers (Table 1).

**Table 1:** **Functional properties of relevance for a potential future biomarker role for some of the proteins used to study MD catheter *in vivo* performance in our porcine brain injury model (16)**.

Protein	MW	Function	Potential biomarker role
Serum albumin	69.7	Osmotic pressure of blood	Breakdown of the blood-brain-barrier
Hemoglobin subunit beta	16.2	Oxygen metabolism	Red blood cell degradation product
Serotransferrin	78.9	Iron transport	Iron ions in the brain parenchyma
Hemoglobin subunit alpha	15.2	Oxygen metabolism	Red blood cell degradation product
Creatine kinase B-type	20.7	Energy metabolism	Used to increase accuracy of S100B as a biomarker
Cystatin C	15.7	Inhibits lysosomal proteinases	Increased autophagal activity
Apolipoprotein A-1	9.18	Lipid transport	Trauma induced membrane remodeling
Alpha-2-HS-glycoprotein (Fetuin-A)	38.4	Ion transport	Possible systemic response to brain injury
Apolipoprotein E	36.7	Lipid transport	Trauma induced membrane remodeling
Complement C3	184	Innate immunity	Innate immune response to brain injury

*MW, molecular weight (kDa); protein function; potential role as a biomarker of acute brain injury. For Uniprot data, see Figure 7. For further discussion, see Ref. (16)*.

### Antibody-based analysis of microdialysate protein biomarkers

There are a number of publications where the temporal response of one to several biomarkers of, e.g., inflammation has been studied in patients with acute brain injury using MD and ELISA-based analyses (2, 3, 5–9). These pioneering studies have begun to unravel the complex features of the inflammatory cascade in acute human brain injury. The limitations are the rather large sample volumes required, resulting in poor temporal resolution, as well as a low number of biomarkers that can be measured in the same sample.

### Multiplex microdialysate protein biomarker analysis

The Luminex technology opens up for new possibilities to monitor complex secondary injury cascades, such as inflammation, directly in the injured human brain by analyzing a larger number of biomarkers in relatively small MD samples. Intriguing studies by Helmy et al. (5, 10) on multiple inflammatory protein biomarkers (measured in duplicate 25 μL samples using a Milliplex MultiAnalyte 42 analyte kit; Millipore, St Charles, MI, USA) further demonstrated the complexity of the temporal inflammatory biomarker patterns in NIC patients with severe diffuse TBI. It was also demonstrated that about half of the 42 biomarkers appeared in higher concentration in MD samples than in arterial plasma suggesting intracerebral biomarker production (5). The data support the concept that the innate immune system of the brain is activated early after TBI, previously developed based on clinical and experimental studies showing, e.g., higher cytokine levels in human CSF compared to blood (59). This makes MD an attractive focal sampling method for many biomarkers, such as cytokines, chemokines, and neurotrophic factors, as a complement to traditional global biomarker analysis in ventricular CSF. The advantage of MD sampling is that it catches the inflammatory mediators in the interstitial space at the site of their cellular actions, also avoiding dilution of the biomarker signals in CSF. In addition, the MD work (5, 10) strongly supports the potential of multiplex analytical methodology as tools to study complex secondary injury cascades directly in the human brain. However, a limitation is the time resolution, which is currently 6 h with the standard MD set up widely used in the NIC setting.

#### Proximity ligation technology

To improve analytical sensitivity and precision, we are collaborating with the Uppsala Berzelii Technology Centre for Neurodiagnostics (www.berzelii.uu.se) to use proximity ligation assay (PLA) technology (60) to apply multiplex panels of biomarkers of brain injury and inflammation in MD samples from TBI patients. Because of the superior sensitivity of the PLA technology compared to conventional sandwich assays (61), we assume that this methodology will have a significantly better time resolution. Another advantage with the Berzelii Centre collaboration is that the PLA multiplex panels can be continuously modified locally as our knowledge of clinically useful biomarkers evolves. Preliminary results support the feasibility of this approach. Pilot data show that modern multiplex PLA panels, based on the new generation Proximity Extension Assay (PEA) technology (Olink Bioscience, Uppsala, Sweden) can measure about 80 biomarkers, including inflammatory mediators, in very small (1 μL) MD samples from TBI patients (unpublished data not shown). This emerging technology may become a powerful tool for biomarker discovery with the potential of analyzing 400–500 biomarkers in hourly MD samples (and in CSF samples) allowing for temporal mapping of individual biomarker profiles as well as complex temporal biomarker patterns.

## Future Perspectives

Our working hypothesis is that the combination of the refined MD method and the new generation PEA panels of multiplex biomarker panels will provide a unique possibility for future studies of the secondary injury cascades following acute human brain injury, paving the way to a better understanding of the complex pathobiology involved and the identification of novel biomarkers and targets for therapeutic intervention. Finally, dedicated smaller bed side PEA panels including the most valuable biomarkers may prove to be useful for individual patient monitoring and tailored treatment in the future NIC setting.

## Conflict of Interest Statement

The authors declare that the research was conducted in the absence of any commercial or financial relationships that could be construed as a potential conflict of interest.
